# 200. Prospective Evaluation of the GenMark Dx ePlex^®^ Blood Culture Identification Gram Positive Panel

**DOI:** 10.1093/ofid/ofab466.402

**Published:** 2021-12-04

**Authors:** Jeremy Meeder, Derek Moates, Hannah Pierce, Jamie Hutchinson, Cameron White, Pia Cumagun, Rachael A Lee, Todd P McCarty, Sixto M Leal

**Affiliations:** 1 University of Alabama at Birmingham, Birmingham, Alabama; 2 University of Alabama at Birmingham; Birmingham VA Medical Center, Birmingham, Alabama

## Abstract

**Background:**

The ePlex BCID Gram-Positive (GP) panel utilizes electrowetting technology to detect the most common causes of GP bacteremia (20 targets) and 4 antimicrobial resistance genes in positive blood culture bottles. Rapid detection of intrinsic vancomycin resistance and acquired resistance genes (*mecA, mecC, vanA, vanB*) enables early optimization of antimicrobial therapy whereas early detection of common contaminants decreases unnecessary antibiotic utilization and hospitalizations.

**Methods:**

In this prospective study, we evaluated the performance of the BCID-GP panel compared to traditional standard of care culture and susceptibility testing with organism identification using the BioMerieux Vitek MS Matrix Assisted Laser Desorption Ionization (MALDI) Time of Flight mass spectrometry. Samples submitted for standard of care testing in Biomerieux BacT/Alert resin FA/FN blood culture bottles on the BacT/Alert VIRTUO automated blood culture system with GP bacteria on direct exam (n=100) were included.

**Results:**

All GP bacteria were represented on the BCID-GP panel, most tests 97/100 (97%) yielded valid results, 53 common skin contaminants (50 coagulase negative staphylococci (CNS), 2 *Bacillus*, 1 *Corynebacterium*) were identified, and 7/7 coinfections with Gram negative (GN) bacteria were detected by the Pan GN target and identified by the BCID-GN panel. Discordant analyses revealed a positive percent agreement (PPA) of 96/97 (99%) with 1 false negative CNS and a negative percent agreement (NPA) of 92/97 (94.8%) with 5 false positives for either *S. epidermidis* or *Corynebacterium*. Detection of *vanA* yielded a PPA of 4/4 and NPA of 9/9. *mecA* gene detection exhibited a PPA of 14/14 and NPA of 14/14 for *S. aureus* and a PPA of 31/32 (97%) and NPA of 16/16 for coagulase negative staphylococci with 1 false negative methicillin resistant *S. epidermidis*.

**Conclusion:**

Detection of acquired vancomycin resistance (n=4) and absence of *mecA* gene detection in *Staphylococcus* species (n=30) represent opportunities for early optimization of antimicrobial therapy in 34/100 (34%) of samples. The BCID-GP panel provides rapid accurate detection of resistant isolates and common contaminants enabling high quality data driven optimization of antimicrobial therapy.

**Disclosures:**

**Todd P. McCarty, MD**, **Cidara** (Grant/Research Support)**GenMark** (Grant/Research Support, Other Financial or Material Support, Honoraria for Research Presentation)**T2 Biosystems** (Consultant) **Sixto M. Leal, Jr., MD, PhD**, **Abnova** (Grant/Research Support)**AltImmune** (Grant/Research Support)**Amplyx Pharmaceuticals** (Grant/Research Support)**Astellas Pharmaceuticals** (Grant/Research Support)**CNINE Dx** (Grant/Research Support)**GenMark Diagnostics** (Grant/Research Support, Other Financial or Material Support, Honoraria- Research Presentation)**IHMA** (Grant/Research Support)**IMMY Dx** (Grant/Research Support)**JMI/Sentry** (Grant/Research Support)**mFluiDx Dx** (Grant/Research Support)**SpeeDx Dx** (Grant/Research Support)**Tetraphase Pharmaceuticals** (Grant/Research Support)

